# QTL Mapping of a High Protein Digestibility Trait in *Sorghum bicolor*

**DOI:** 10.1155/2009/471853

**Published:** 2009-07-07

**Authors:** Jennifer A. Winn, R. Esten Mason, Adriana L. Robbins, William L. Rooney, Dirk B. Hays

**Affiliations:** Department of Soil and Crop Sciences, Institute for Plant Genomics & Biotechnology, Texas A&M University, 2474 TAMU, College Station, TX 77843, USA

## Abstract

Compared with other cereal grains, *Sorghum bicolor* shows lower protein digestibility. The low digestibility is thought to result from disulfide cross linking in the *β*- and *γ*-kafirins. In contrast, the single recessive high digestibility/high lysine content (HD) mutation which confers greater grain digestibility exists in sorghum that is thought to result from reduced accumulation of *γ*-kafirin that allows greater access to the high digestible *α*-kafarin fraction. In an effort to both clearly define the molecular basis for the HD trait and develop tools to improve the introgression of this difficult-to-screen trait, this study focuses on mapping the QTLs linked to this trait. While the HD trait has been defined as a single recessive gene, our results uncovered that two major QTLs on chromosome 1 are associated with protein digestibility—one QTL (locus 1 from the HD parent) unfavorably affects digestibility and one QTL (locus 2 from the HD parent) only 20 cM away favorably affects digestibility. A contrast analysis between genotypic groups at these two loci shows that a higher level of protein digestibility may be obtained when this linkage in repulsion is broken and favorable alleles are allowed to recombine.

## 1. Introduction

Millions of people throughout the world—including Africa, Asia, and other semiarid regions—depend on sorghum as a staple crop. In many households, sorghum is the primary source for energy, protein, vitamins, and mineral [1]. As the fifth most abundant crop worldwide [2] and the third most economically important crop in the US [3], sorghum plays a huge role on the world market as a means of livelihood for millions of subsistence farmers and as an important part of food security [4]. Furthermore, sorghum is used as an important feed source, particularly in developed countries such as the US. Worldwide, 31 million tons, or 48% of all sorghum grown, are used for livestock feed [5]. 

Despite its extensive use, sorghum is low in protein digestibility. When cooked, only 46% of the total protein found in sorghum is digestible, as compared with 81% in wheat, 73% in maize, and 66% in rice [6, 7].

Several theories have been proposed as to the cause of sorghum's reduced digestibility [4] but it is most widely accepted that structural interactions between the *α*-, *β*-, and *γ*-kafirins (prolamin-type protein storage molecules in the grain endosperm) play a critical role in protein digestibility. 

At the periphery of the spherical protein bodies are *β*- and *γ*-kafirins, making up a combined 20% of kafirin content, most of which are *γ*-kafirins. Both *β*- and *γ*-kafirins have high concentrations of the amino acid cysteine. Of total amino acid content in *β*-kafirins, 5% is cysteine; in *γ*-kafirins, 7% is cysteine [8]. *α*-kafirins are found at the interior of the protein body and make up ≈80% of kafirin content and ≈60–70% of total protein content within the grain [9].

The high cysteine concentration in *β*- and *γ*-kafirins causes extensive disulfide cross linking when cooked. As a result, the kafirins (particularly the *γ*-kafirins) form polymers that create a tightly bound structure encapsulating the *α*-kafirins. The cross linked *γ*-kafirin barrier is resistant to proteolytic enzymes, which in turn causes the normally highly-digestible *α*-kafirins to be inaccessible to proteolytic enzymes [4]. When sorghum is cooked with a reducing agent, such as 2-mercaptoethanol—to break disulfide cross linking—protein digestibility is higher than when cooked in water alone, verifying the observation that it is disulfide cross linking that causes inhibited digestibility [9, 10].

A highly digestible (HD) sorghum lines derived from a high-lysine chemical mutant (P721 Opaque, also known as P721Q) [11, 12] have been found to have ≈10–15% higher protein digestibility when uncooked and ≈25% higher digestibility when cooked. More specifically, *α*-kafirin digestibility increased to ≈90–95% following pepsin digestion (≈45–60% digestibility in normal cooked lines) [11].

The cause for the higher digestibility in specific lines is thought to be due to rearrangement of the kafirins, particularly the *γ*-kafirins, located at the exterior of the protein bodies. Instead of the *γ*-kafirins being located around the periphery of a protein body as they are in normal lines, highly digestible lines possess *γ*-kafirins that are found only in pockets of folds within the total protein body [13].

As a result of the physiological changes of the HD endosperm protein body structure, the interior *α*-kafirins are exposed, making them susceptible to proteolytic enzymes. Furthermore, with the invaginated structure of the highly digestible lines, there is more total surface area available for hydrolysis by digestive enzymes.

This research study aims to analyze the genes controlling protein digestibility (likely as a result of kafirin rearrangements) using molecular markers. Marker-assisted selection (MAS) is becoming an increasingly useful tool to breeders, and it is our goal to add them to the toolbox by providing a genetic analysis of sorghum protein digestibility.

## 2. Materials and Methods

### 2.1. Seed Lines

Seed in the F_4_ generation was obtained from 277 recombinant inbred lines resulting from a cross between the highly digestible line P850029 and the wild type line Sureno. Many individuals had sib lines used in the study. An individual and its sib (i.e., two F_3:4_ lines) were from the same parents and grown in the same location. Sib lines were valuable because they could approximate a replication in statistical analyses since seed from multiple years, replications, and environments was not available.

### 2.2. Protein Digestion

The amount of 50 mg of seed from each sample was ground and added to 1 mL pepsin solution (20 mg pepsin/mL 0.1 M KH_2_PO_4_, pH 2) in a 1.5 mL tube. The mixture was vortexed and incubated with shaking at 37°C, 2 hours. 100 *μ*L of 2N NaOH was added and samples were centrifuged at RCF = 30,733 × g, 10 minutes. Supernatants were discarded and samples were resuspended in 1 mL 0.1M potassium phosphate buffer, pH 7. The samples were pelleted (RCF = 30,733 × g), washed with 1 mL ddH_2_O, and repelleted.

To extract proteins, the pellets after pepsin digestion were incubated at 37°C in a water bath with 0.5 mL extraction buffer (0.0125 M sodium tetraborate pH 10; 1% SDS (sodium dodecyl sulfate) W/V; 2% mercaptoethanol). Samples were centrifuged at RCF = 15,680 × g for 10 minutes at 4°C. From the middle layer of supernatants, 200 *μ*L was transferred to a clean 1.5 mL tube to continue to the turbidity assay.

### 2.3. Turbidity Assay

25 *μ*L per sample was transferred to another new 1.5 mL tube, to which 1 mL of H_2_O and 200 *μ*L of 72% trichloroacetic acid (TCA) were added. A blanking solution was prepared with 25 *μ*L protein extraction buffer, 1 mL of H_2_O, and 200 *μ*L of 72% TCA. The spectrophotometer was set to record turbidity at 562 nm. Readings were taken at 15 and 30 minutes after adding TCA to the protein solution.

Originally, measurements were also taken at 45 and 60 minutes, as well, but turbidity readings across time were not found to vary. The samples with the highest and lowest turbidity readings were rerun through the entire process (from the flour stage) to ensure that the measurements were reliable in determining whether each sample could be considered LD or HD. To ensure reliability in readings, samples with any two readings differing by more than a value of 0.4 were eliminated from subsequent analyses. Averages of all measurements were taken for each sample, which were then used to rank the lines in terms of digestibility—the averages are what will from now on be referred to as the turbidity values.

### 2.4. DNA Extraction

Each seed line (277 lines total, including parents) was grown out for 15 days, and plant tissue was collected. DNA was extracted based on the procedure described by Dellaporta et al. [14]. Each sample was diluted with ddH_2_O to a final concentration of 10 ng/*μ*L.

### 2.5. Bulked Segregant Analysis

Bulked segregant pools were created using the procedure developed by Michelmore et al. [15]. The highly digestible (HD) DNA bulk was pooled from the five lines showing the lowest turbidity values. Each line was added in equal concentration, and the final bulked DNA was diluted to 10 ng/uL. Similarly, the lowly digestible (LD) DNA bulk was pooled from the five lines showing the highest turbidity averages.

### 2.6. Molecular Marker Analysis

Next, 355 SSR primers were screened for polymorphisms using DNA from the 2 parents. PCR reactions were set up as follows for one 10 *μ*L reaction: 1 *μ*L buffer, 0.1 *μ*L dNTPs (10 mM), 1.5 *μ*L each of forward and reverse primer (2 *μ*M), 0.5 *μ*L MgCl_2_, 0.1 *μ*L BSA, 2 *μ*L DNA (10 ng/*μ*L), 0.1 *μ*L Taq Polymerase, and 3.2 *μ*L H_2_O. The PCR conditions were 94°C for 5 minutes; 40 cycles of 1 minute at 94°C, 1 minute at the primer annealing temperature, 1 minute at 72°C; 10 minutes at 72°C; hold at 4°C. All PCR products were run on 3% agarose gels for 2 hours. Approximately 100 of the 300 original SSR primers showed polymorphisms between the parents. The 100 primers were then run using the 2 DNA bulks (HD and LD), and the number of primers found to be polymorphic between the bulks was 8 (see Appendix B for primer information). Finally, the 8 primers were run across 70 randomly chosen individuals from the population. Only one individual from each sib pair was included in primer analyses to prevent over representation of a genotype.

Genotypes from each individual were scored as being one of the 2 parental genotypes or as being heterozygous. Individual genotypes and corresponding phenotypes (as evaluated by turbidity values) were entered into MapMaker 3.0 and QTL Cartographer. A QTL map and marker linkage data were generated using segregation data. Distances are calculated using the Kosambi function [16]. Measurements are given in centiMorgans (cM).

### 2.7. Contrast Analysis

A contrast analysis for the markers most closely linked to the QTL regions was carried out using ANOVA in SPSS for Mac. Lines were grouped into four genotypic classes (AA, BB, AB, BA) based on genotype and the mean turbidity values for each genotypic class were calculated. For ANOVA, genotype was treated as an independent variable having a fixed effect and turbidity values were treated as dependent variables. Two post-hoc analyses, Fisher LSD and Tukey HSD, were used to identify significant differences between groups.

## 3. Results

**Figure 1:**
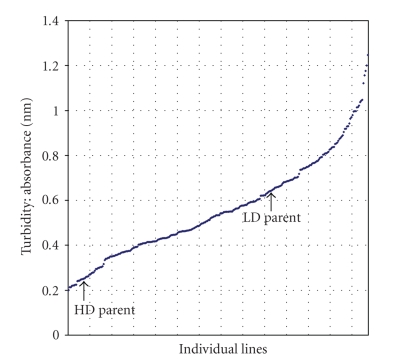
Turbidity assay results. The turbidity of each sample at 562 nm correlates to protein digestibility. Samples with high protein digestibility are represented by low turbidity values. The distribution of turbidity values is surprisingly gradual (not step-wise). It is also interesting to note that there are many lines showing transgressive segregation.

The results from the turbidity assay show a gradual increase in turbidity across the population of 277 individuals, including parents and sibs. If protein digestibility was truly controlled by a single, simply inherited gene, as was previously theorized, the turbidity assay should show a step-wise distribution between LD, heterozygous, and HD individuals (Figure 1). As expected, the HD parent falls in the range of low turbidity, while the LD parent has higher turbidity. It is interesting to note that there are many individuals with turbidity values higher than the LD parent. It is hypothesized that transgressive segregation or highly favorable multiallelic combinations could be contributing to the phenotypes of these extreme individuals.

**Figure 2:**
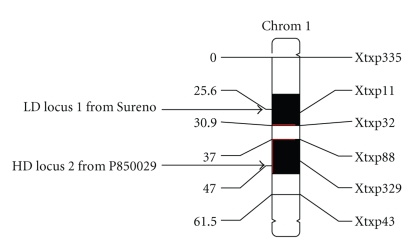
Linkage map of primers segregating with protein digestibility trait. Shaded areas are significant QTLs. The region—Locus 1—near Xtxp11 is the LD locus and unfavorably impacts digestibility, while the region—Locus 2—near Xtxp329 is the HD locus and favorably impacts digestibility. Distances are given in centiMorgans (cM).

Mapmaker 3.0/QTL Cartographer analysis of phenotypic values was performed (*n* = 70, mean = 0.6109; standard deviation = 0.2187), and genotypes were found to be highly significant (*α* = 0.001), using sib lines as a blocking effect to approximate replications. Eight molecular markers were shown to be polymorphic between the parental lines and between the bulked groups. The eight markers were scored against 70 random individual lines, and six markers were shown to segregate together (Figure 2). According to previous marker analyses of the sorghum genome [17, 18] the six markers are located on Chromosome 1.

**Figure 3:**
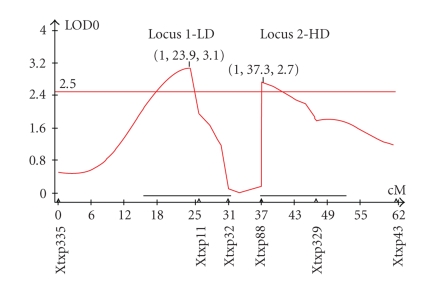
QTL positions with LOD scores. Two QTLs were found to associate with high protein digestibility. The QTL on the left (Locus 1) is the LD locus and contributes unfavorably to digestibility, while the QTL located on the right (Locus 2) is the HD locus and contributes favorably. LOD scores over 2.5 were considered significant.

**Figure 4:**
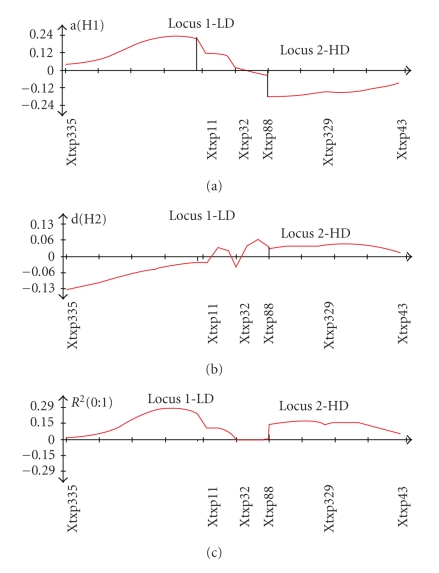
The additive, dominance, and R2 effects of Locus 1—near Xtxp11 which is the LD locus that unfavorably impacts digestibility, while the region—Locus 2—near Xtxp329 is the HD locus that favorably impacts digestibility. (a) Additive effect of QTLs: units are phenotypic values of the turbidity assay. The QTL on the left decreases digestibility, thus it is shown to have a positive additive affect—it increases turbidity; the QTL on the right works conversely. (b) Dominance effect of QTLs: units are phenotypic values of the turbidity assay. The QTL on the left (Locus 1) has a stronger dominance effect than the QTL on the right (Locus 2). This leads to the left QTL contributing more to overall phenotypic variation. (c) R^2^ for the QTLs: the QTL on the left (Locus 1) correlates stronger to phenotypic values—turbidity averages—than the QTL on the right (Locus 2). Graphs show the effects of the B allele from the HD parent.

Two major QTLs (Figure 3) were found to be significant at LOD > 2.5. One major QTL (which will now be referred to as “Locus 1”) occurs near marker Xtxp11 and shows an LOD score of 3.1. The QTL at this locus is surprising in that it displays additive and dominance effects that act unfavorably in terms of protein digestibility, as shown in Figures 4(a)  and 4(b). The percent of phenotypic variation (R^2^) explained by the alleles at this locus accounts for approximately 29% of the total variation seen in Figure 4(c).

Conversely, only approximately 20 cM away lays a second QTL (which will now be referred to as “Locus 2”) located between Xtxp88 and Xtxp329. This locus has an LOD score of 2.7 and an R^2^ value of 18%. As opposed to the first QTL, this locus favorably affects protein digestibility and is likely the HD locus. That is, an increase in favorable alleles at this locus serves to increase protein digestibility (decreases turbidity value). Although two significant QTLs were found, no individual marker was found to be significant (Table 1).

A contrast analysis was calculated using the two markers segregating closest to the two QTLs—markers Xtxp11 and Xtxp329. In the analysis, recombinant inbred lines were grouped and labeled according to their alleles at Loci 1 and 2 and the mean turbidity value was calculated for all lines within each genotypic group. For instance, “AB” indicates that individuals in this genotypic group had the genotype of parental type A (the LD line Sureno) at Locus 1 (the LD locus) and the genotype of parental type B (the HD line 9850029) at Locus 2 (the HD locus). Genotypic groups included in the analysis were AA, AB, BA, and BB. Only homozygous lines for each locus were used in the contrast analysis. The goal of the contrast analysis was to determine whether the four genotypic groups were correlated with phenotypic value (turbidity average) and whether significant differences in turbidity could be detected between groups.

An ANOVA indicated that there was a significant difference (*α* = 0.05) in phenotypic values between at least two of the groups. Two post-hoc analyses, Fisher LSD and Tukey HSD, were used to calculate which of the phenotypes showed significant differences in phenotypic values, with phenotypic group BA showing significant differences from the other three groups (*α* = 0.05).

The results of these analyses indicate that the highest protein digestibility is found in the AB genotypic group. That is, individuals with the parental type A from the LD-parent allele at Locus 1 and parental type B from the HD-parent allele at Locus 2 have higher average levels of protein digestibility. The favorable alleles at the two loci contributing to protein digestibility are segregating in repulsion in the parental lines. This can explain why the two parental phenotypic values are not as different from each other as expected; each possesses favorable alleles at one locus and unfavorable alleles at the other. The two favorable alleles linked in repulsion also explain the transgressive segregation shown in the phenotypic values of the whole population. The AB genotypic group contains both of the favorable alleles, resulting in higher protein digestibility compared to the other genotypic classes. Conversely, the lowest digestibility occurs in the BA genotypic group, which possesses both of the unfavorable alleles for digestibility. 

When recombination occurs to break the linkage in repulsion, phenotypic values can be expected to surpass either parent's value. A slight gain in digestibility may be obtained by breaking the linkage group in the parental lines to acquire favorable alleles segregating together, as seen in the AB genotypic group. Whether the gain in digestibility will confer a biologically significant increase in digestibility must be evaluated within the context of a breeding program. Furthermore, it should be noted that, as with any genetic linkage map, the results might only be applicable to the population and environment studied.

## 4. Discussion

The turbidity assay data (Figure 1) indicates that the phenotypic value of protein digestibility does not occur in a step-wise fashion, but rather as a gradient. When the broad distribution for phenotypes is viewed in light of the QTL analysis, it becomes evident that there are complex genetic interactions controlling protein digestibility, possibly with many genetic and molecular modifiers controlling phenotype. Furthermore, because the two significant QTLs are essentially working against each other in terms of overall effect on protein digestibility (as analyzed by the turbidity assay), it is likely that each locus has a greater influence on phenotypic variation than was calculated. Since the QTLs are opposed and closely linked, QTL Cartographer may have underestimated each of the QTLs' significances. Still, summing the two QTLs contributes to 47% of total phenotypic variation, which is a very reasonable percentage. According to Tanksley (1993), the average percent of phenotypic variation explained by QTLs in experimental studies is 30–40%.

Although protein digestibility was originally thought to be a simply inherited, recessive trait controlled by a single gene, this is not likely the case—the gradient of phenotypic values is probably caused by various alleles at multiple loci working favorably or unfavorably to influence protein digestibility. A hypothesis emerges that protein digestibility is controlled by two distinct, side-by-side regions on Chromosome 1—Locus 1 (an inhibitor of digestibility) and Locus 2 (a promoter of digestibility).

There are clearly many possibilities as to how the gene regions affect protein digestibility (for which further research is needed), but the QTLs found in this study may in the meantime prove beneficial to breeders using MAS.

## Figures and Tables

**Figure 5:**
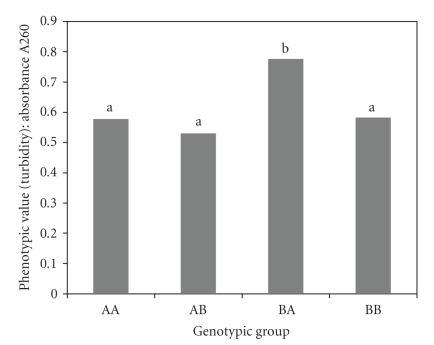
Phenotypic averages of the genotypic groups. Genotypic group “BA” represents individuals having parental type B (the HD line P850029) at Locus 1 and parental type A (the LD line Sureno) at Locus 2. Phenotypic values are represented by turbidity averages. Letters above the bars indicate significant difference groups where genotypic groups with the same letter are not significantly different (*α* = 0.05).

**Table 1:** Summary of marker segregation. The number of individuals with informative results is given, along with the LOD score according to the QTL distribution and the probability that each marker segregates independently of the protein digestibility trait (QTL Cartographer Single Marker Analysis).

	Chromosome 1		
Name	*n*	LOD	pr(F)
Xtxp335	70	0.6	0.710
Xtxp11	69	2.1	0.144
Xtxp32	68	0.1	0.478
Xtxp88	65	1.5	0.656
Xtxp329	70	1.8	0.361
Xtxp43	64	1.2	0.521

## Abbreviations

HD: High digestible
LD: Low digestible
LOD: Likelihood of odds
MAS: Marker-assisted selection
PCR: Polymerase chain reaction
QTL: Quantitative trait loci
SSR: Simple sequence repeat (microsatellite) marker
